# Animal Behavior Frozen in Time: Gregarious Behavior of Early Jurassic Lobsters within an Ammonoid Body Chamber

**DOI:** 10.1371/journal.pone.0031893

**Published:** 2012-03-07

**Authors:** Adiël A. Klompmaker, René H. B. Fraaije

**Affiliations:** 1 Department of Geology, Kent State University, Kent, Ohio, United States of America; 2 Oertijdmuseum De Groene Poort, Boxtel, The Netherlands; University of Arizona, United States of America

## Abstract

Direct animal behavior can be inferred from the fossil record only in exceptional circumstances. The exceptional mode of preservation of ammonoid shells in the Posidonia Shale (Lower Jurassic, lower Toarcian) of Dotternhausen in southern Germany, with only the organic periostracum preserved, provides an excellent opportunity to observe the contents of the ammonoid body chamber because this periostracum is translucent. Here, we report upon three delicate lobsters preserved within a compressed ammonoid specimen of *Harpoceras falciferum*. We attempt to explain this gregarious behavior. The three lobsters were studied using standard microscopy under low angle light. The lobsters belong to the extinct family of the Eryonidae; further identification was not possible. The organic material of the three small lobsters is preserved more than halfway into the ammonoid body chamber. The lobsters are closely spaced and are positioned with their tails oriented toward each other. The specimens are interpreted to represent corpses rather than molts. The lobsters probably sought shelter in preparation for molting or against predators such as fish that were present in Dotternhausen. Alternatively, the soft tissue of the ammonoid may have been a source of food that attracted the lobsters, or it may have served as a long-term residency for the lobsters (inquilinism). The lobsters represent the oldest known example of gregariousness amongst lobsters and decapods in the fossil record. Gregarious behavior in lobsters, also known for extant lobsters, thus developed earlier in earth's history than previously known. Moreover, this is one of the oldest known examples of decapod crustaceans preserved within cephalopod shells.

## Introduction

Gregarious behavior of organisms is known to have numerous advantages such as resource exploitation, mating success, environmental modification, and reduction of the risk of predation (see [1] and references therein). This behavior is known for modern marine arthropods such as brachyurans (e.g. [2]), anomurans (e.g. [3]), and macrurans (e.g. [4], [1]). Gregarious sheltering was reported for the palinurid *Scyllarides latus* [5] and appears to be common for clawless lobsters ([6] and references therein).

Gregarious behavior is also known from the fossil record (e.g. [7] for trilobites and references therein). Examples of this behavior preserved within empty mollusk shells from the fossil record are extremely rare. However, those examples known, show that mollusk shells were (temporarily) inhabited by numerous types of organisms. In the Paleozoic, trilobites inhabited cephalopod shells (e.g. [8], [9], [10]). For example, three examples with more than one individual of the same trilobite species within a cephalopod shell are known [8]. These occurrences seem to support the idea that trilobites assembled in monospecific clusters for molting prior to *en masse* copulation. Nice examples from the Late Cretaceous of Kansas (USA) are flocks of fish preserved within the shells of large inoceramid bivalves [11], [12]. At least five different fish genera occurring in groups of up to 104 specimens were preserved within the large valves of the *Platyceramus platinus* [11], [12]. Another example of fossilized *in situ* cave dwellers are small heteromorph ammonoids preserved within large pachidiscid ammonoid shells from the Late Cretaceous of Japan [13]. Small ammonoids that used a larger shell as a shelter are also known [14]. Furthermore, Triassic ophiuroids were reported to hide within a ceratite ammonoid, possibly for inquiline purposes and to brood [15]. Lastly, Upper Cretacous (Campanian) echinoids sought shelter in an ammonoid found in northern Germany [16].

Gregarious behavior of lobsters may be known from the fossil record. Tsujita [17] noted that four concretions of the Upper Cretaceous (upper Campanian-lower Maastrichtian) Bearpaw Formation in Alberta (Canada) contained two specimens each of the lobster *Palaeonephrops browni*. He interpreted these concretions to represent burrows. If this interpretation is correct, these concretions could represent gregarious behavior of lobsters. Concretions of the same formation but from Montana (USA) containing two lobster specimens were already known [18]. The only other example of possible gregarious behavior in Mesozoic lobsters known to us is from the Lower Jurassic (Toarcian) of Greenland [19]. Concretions containing specimens of *Glyphea rosenkrantzi* were found *in situ* in burrows known as *Thalassinoides* in the upper part of the Ostreaelv Formation (upper Toarcian) [19], [20]. Although none of the concretions was mentioned to contain two lobsters [19], [21], an illustration in [19] (their Fig. 6) suggests that several lobsters lived in the same burrow system.

Gregarious behavior from the fossil record is also recorded for shrimp. Numerous papers report on two or more callianassid claws preserved in burrows (e.g. [22], [23], [24], [25], [26], [27], [28]). The claws may represent more than one individual. The oldest known examples are from the Late Cretaceous [22], [23], [26]. Numerous callianassids present in one burrow system are also known from the present [29], [30]. We are unaware of gregarious behavior of non-lobster decapods prior to the Cretaceous. The aim of this paper is to report upon the oldest example of definite gregarious behavior of fossil lobsters and decapods.

### Geological setting

**Figure 1:**
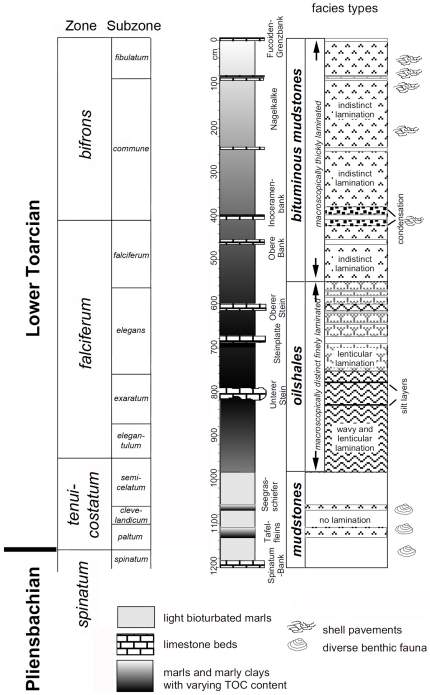
Stratigraphical and biostratigraphical profile of the lower Toarcian Posidonia Shale of Dotternhausen (modified after[34] with permission given by SEPM (Society for Sedimentary Geology)). The specimen was collected near the Inoceramenbank, at 400 cm depth.

**Figure 2:**
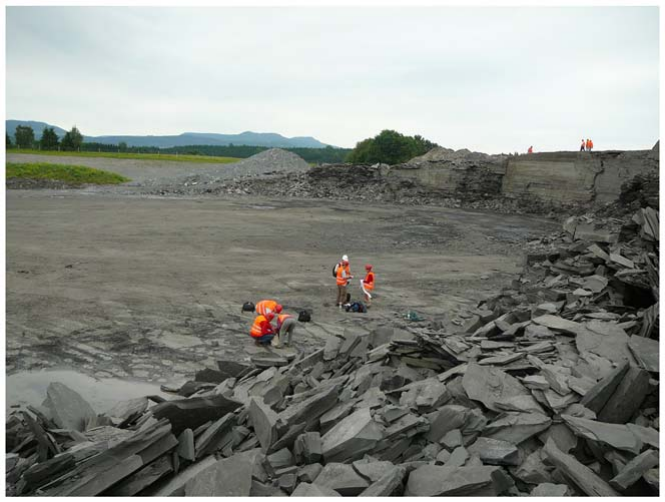
Impression of the Posidonia Shale in the quarry in Dotternhausen.

The lower Toarcian Posidonia Shale is famous for its excellent preservation of marine fossils and its high amount of organic matter. Both quality of preservation and accumulation of organic matter have been explained by permanent anoxic bottom waters known as the stagnant basin model [31]. In Dotternhausen near Balingen, 70 km southwest of Holzmaden in southwestern Germany, the Posidonia Shale is quarried by the Holcim (Süddeutschland) GmbH for cement production (Figures 1, 2).

High-resolution geochemical, sedimentological and paleoecological investigations of the exposed section in Dotternhausen showed that oxygen availability was variable and ranged from short oxygenated periods to longer-term anoxia [32]. Indeed, benthic organisms such as bivalves, brachiopods, lobsters, and serpulids are known from the Posidonia Shale at Dotternhausen [33]. The variations in oxygen content were probably induced by a strong meridional atmospheric circulation system with pronounced seasonal changes of prevailing trade- and monsoon-wind systems [32]. During the monsoon-influenced summer months, a stratified water column with anoxic conditions developed below the halocline. During the winter months, a saline circulation system brought oxygen to the benthic environment, favoring temporary benthic colonization, especially during times of relative sea level highstand [32].

The sediments at Dotternhausen show very euxinic conditions during the early *falciferum* Zone (oil shale); more aerated bottom waters established during the late *falciferum* Zone (bituminous mudstone) [34]. Just below and above the ‘Inoceramenbank’ in the Dotternhausen section (see Fig. 1), long-term aerated bottom waters were proposed to exist [34]. This is exactly that part of the stratigraphic column from which several ammonoids with contents in their body chambers have been found [35]. The phragmocones of the ammonoids are compressed to a thickness of only 1–2 mm, because of the very rapid sedimentation rate at the time of deposition of the Posidonia Shale [32]. The calcareous shell layers are dissolved, but the periostracum is preserved as a very thin, golden brown, translucent coating. This allows for exploring the inside of the body chamber. Nearly 4% of the relatively large body chambers of adult *Harpoceras falciferum* macroconchs contain distinctive crop content, mostly pereiopods of small decapod crustaceans and small aptychi [35]. The inquiline use of large body chambers of adult *Harpoceras falciferum* macroconchs by several fish of the genus *Pholidophorus* and the lobster *Palaeastacus* sp. was previously noted [36], [37]. The example presented herein of Early Jurassic eryonid lobsters preserved in the ammonoid *Harpoceras falciferum* macroconch was collected from the same stratigraphic level.

## Results

### Location of the lobsters within the ammonoid

**Figure 3:**
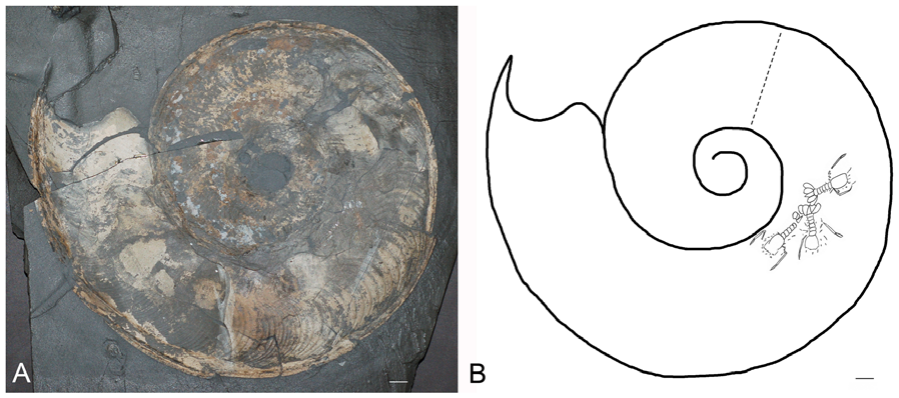
A view of the compressed specimen of the ammonoid *Harpoceras falciferum* containing the three lobsters. (A) photo and (B) line drawing. The dashed line indicates the transition from the body chamber to the phragmocone. The scale bars represent 10 mm.

**Figure 4:**
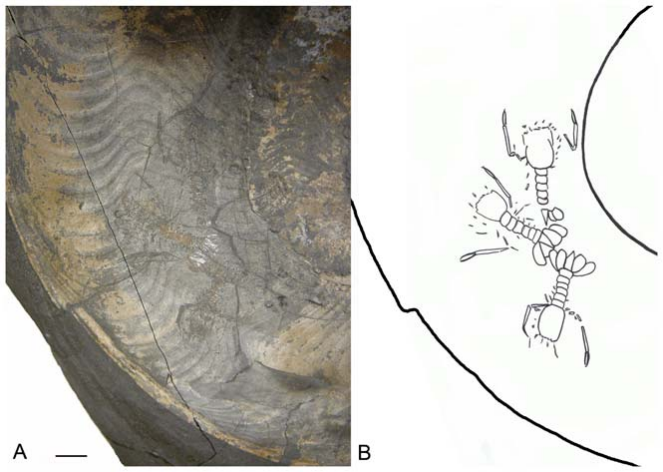
Detailed view of the lobsters in the ammonoid body chamber. (A) photo and (B) line drawing. The scale bar represents 10 mm.

The three lobsters are found within a body chamber of the Toarcian ammonoid *Harpoceras falciferum* from Dotternhausen, Germany. The diameter of the ammonoid shell is 230 mm and the aptychi are missing. Because the ammonoid is two-dimensionally compressed with only the golden brown translucent periostracum preserved, it can be inferred that the lobsters are located inside the body chamber and not on top or below the ammonoid. As for the ammonoid, only the organic remains of the lobsters are preserved. The lobsters are located within the body chamber in the outermost whorl. The central one of the three lobsters is approximately 170° from the aperture and about 90° from the last septum (Figure 3). The other two lobsters are very close to it; approximately 10° from the central one, with their tails centrally and cephalothoraxes radially directed (Figure 4).

### Description of the lobsters

**Figure 5:**
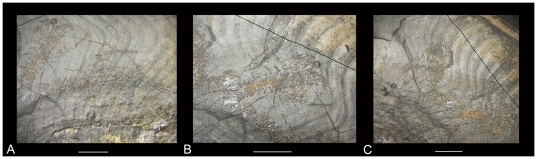
Close-up photos of the three lobsters. ‘A’ represents the lobster that is closest to the aperture, ‘B’ is the middle specimen, and ‘C’ is the specimen closest to the phragmocone. The scale bars are 10 mm.

The cephalothoraxes of the three lobsters are longer than wide; the outline is subcircular to rectangular (Figure 5). None of the cephalothoraxes exhibits a branchiocardiac or cervical groove; instead, longitudinal carinae (small ridges) are present. The longitudinal carinae are present on the posterior part of cephalothorax of the lobster closest to the aperture; on the anteriormost part it is accompanied by subparallel carinae on both sides that curve more laterally in the posteriormost part. The central cephalothorax exhibits three longitudinal carinae on the posterior part. The middle carina is located on the longitudinal axis; the second and third carinae are oriented more laterally and parallel the middle carina. These carinae are longer than the middle one, curve more laterally on the posteriormost parts, and do not connect to the posterior rim. The innermost cephalothorax exhibits two longitudinal, long carinae on both posterolateral parts and two smaller, parallel carinae that originate on the posterior edge. The outermost cephalothorax is more elongated than the other two cephalothoraxes; it also shows a narrower front, possibly due to compression/degradation prior to burial.

Five abdominal segments can be observed for the outermost lobster; six abdominal segments are visible in the central and innermost lobsters. The tergum is rectangular. The shape of the epimeres is not well visible. The central and innermost lobsters exhibit isolated, faint traces of an abdominal keel.

Only a part of the telson is visible in the outermost lobster; the uropod is small. The telson of the central lobster is triangular, about twice as long as wide with the apex pointed posteriorly. The oval-shaped endopods are somewhat larger than the similar-shaped exopod; both contain a longitudinal carina on their axes that extend the entire length of the endopod. The endopod is nearly as long as the telson. The outer part of the exopod is less curved than the inner part. No diaeresis is present on the uropods. The tailfan forms a convex shape as a whole. The tailfan of the innermost lobster is identical to the central one.

The first pair of the pereiopods represents the longest pair and they are homochelous. The propodus and dactylus are nearly parallel to the body axis. In the outermost specimen, the anteriormost pereiopods are chelate and the dactylus is located on the outer side of the pereiopod. The dactylus is slightly longer than the anterior part of propodus. The tips of propodus and dactylus curve inward. The anterior part of the propodus and the dactylus of the right first pereiopod are nearly equal in length. The merus exhibits a nearly 55° angle with the propodus and is nearly transversely oriented to the longitudinal axis. The first pairs of pereiopods of the central and innermost specimens resemble those of the outermost specimen. The outermost lobster exibits two antennules and two antennae. The orbits are not preserved in the three specimens. For measurement of the specimens see Table 1.

**Table 1:** Measurements (in mm) of the three lobster specimens.

	length cephalothorax	length abdomen (excl. telson)	max. length telson/uropods
outermost lobster (closets to aperture)	12	11	>4
central	10	10	6
innermost (closest to air chambers)	10	10	6

Their relative dimensions vary somewhat, possibly related to the compression.

### Taxonomic identity of the lobsters

The overall outline and details on the cephalothoraxes and abdomina suggest that the lobster specimens belong to the Eryonoidea. Three families were reported to belong to the Eryonoidea [38]: the Coleiidae, Polychelidae, and Eryonidae. Recently, two families were added to this superfamily [39]: the Tetrachelidae and Palaeopentachelidae.

Karasawa et al. [40] provided an emended diagnosis for the Coleiidae. This family exhibits a cervical and postcervical groove; both are absent in our specimens. Medial and branchial carinae and an abdominal keel, albeit faint, are present in the studied specimens. The exopod exhibits no diaeresis in our specimens, which differs from the Coleiidae. In conclusion, the specimens cannot be assigned to the Coleiidae. The specimens cannot be assigned to the Polychelidae, Tetrachelidae, or Palaeopentachelidae either. The Polychelidae exhibit pronounced cervical grooves and a spinose lateral margin, which is not the case in the studied specimens (see [38]). The Tetrachelidae are different as well because they do exhibit a cervical and branchiocardiac groove, and their telson is rounded posteriorly instead of pointed. The family Palaeopentachelidae [41] exhibits a median-only cervical groove, which is absent in the specimens described here. Also, the occlusal margins of the propodus and dactylus do not exhibit spines, whereas members of the Palaeopentachelidae exhibit these spines [41]. The specimens fit the diagnosis of the Eryonidae [38]. A rectangular outline of the cephalothorax can be observed in the specimens. The cervical groove and longitudinal keels are absent or short for this family. In the specimens, the cervical groove is absent, while longitudinal keels are only observed in the posteriormost part of the cephalothoraxes. As noted in the family diagnosis, the uropods do not exhibit a diaeresis. The other characteristics (well-developed eyes and first four pereiopods chelate) could not be observed in our specimens due to the mode of preservation. Feldmann et al. [42] (p. 405) stated that ‘the Eryonidae have a narrow front and well-defined orbits, and if longitudinal carinae are present, they seem to be confined to the posterior part of the carapace.’ This is consistent with the specimens described herein.

The Eryonidae currently consist of four genera [39]: *Eryon*, *Cycleryon*, *Rosenfeldia*, and *Knebelia*. The uropods are rounded instead of pointed which would exclude *Eryon*. The mode of preservation, where the periostracum of the ammonoid encases the lobsters and obliterates details, does not allow further ascription to the genus and species level.

## Discussion

### Decapods in cephalopods

The presence of decapods in fossil cephalopod shells is known [16], [36], [43], [44], [45], [46]. Table 2 lists Mesozoic and Cenozoic decapods preserved in cephalopods with the exception of hermit crabs preserved in cephalopod shells [47], [48], [49]. These shells in Table 2 contain only one decapod, providing no evidence for gregarious behavior for decapods for these examples. Table 2 shows that the specimens described here are among the oldest decapod crustaceans preserved within cephalopod shells.

**Table 2:** Examples of Mesozoic and Cenozoic decapods preserved within cephalopods.

System	Stage	Cephalopod	Decapod	Country	Source
Jurassic	Toarcian	*Harpoceras falciferum*	three eryonids	Germany	herein
	Toarcian	*Harpoceras* sp.	*Palaeastacus* sp. with coprolites	Germany	[36]
	Kimmeridgian	*Lytoceras* sp.	*Mecochirus* sp.	New Zealand	[45]
	“Portlandian”	Perisphinctid	*Eryma dutertrei*	UK	[36]
	“Portlandian”	*Gravesia gigas*	*Glyphea leionoton*	Germany	[43]
Cretaceous	Cenomanian	*Calycoceras*? sp.	*Diaulax oweni*	UK	[44]
	Turonian	*Vascoceras* sp.	*Meyeria* sp.	Nigeria	Pers. observation
	Turonian	*Cymatoceras* sp.	Callapid	Germany	Pers. observation
	Campanian	*Pachydiscus* sp.	Brachyuran	Germany	[16]
Paleocene	?Thanetian	*Eutrephoceras* sp.	*Glyphithyreus wetherelli*, *Eocarpilius* sp., and *Palaeocarpilius* sp.	Spain	[46]

*Cymatoceras* sp. and *Eutrephoceras* sp. are the only nautiloid shells; the rest are ammonoid shells.

### Transportation and ingestion by the ammonoid

The ammonoid must have died and sunk to the bottom upon which it became available for occupation by benthic organisms such as lobsters. The lobsters from this study most likely used the ammonoid as some kind of shelter and were not washed in by bottom currents, nor were they part of the crop/stomach contents of the ammonoid. Although not all details are visible, the three lobsters appear to be complete or nearly so. A crop/stomach content interpretation is impossible because of this. If the lobsters had been stomach content, smaller pieces of these lobsters would be expected. This was previously observed in ‘food balls’ in *Harpoceras falciferum* from the Toarcian of Dotternhausen containing parts of loose pereiopods, some abdomina and telsons from decapods, or aptychi from small ammonoids [35]. Not a single piece of carapace could be recognized within the dozens of reported food balls.

The completeness of the lobsters and the presence of more than one individual in virtually the same spot within the ammonoid, and their radial tail to tail preservation, seem to exclude the possibility of transportation into the body chamber by bottom currents. Moreover, cephalopod apertures tend to orient themselves down-current [50], [51], [52].

Mundlos [53] proposed a model for sediment infill of ceratite ammonoids. In the early phases of infill, water enters the body chamber along the ventral side and exits the shell through the phragmocone and dorsal side of the body chamber. In the central portion between the ventral and dorsal side, some flow exists from the ventral to the dorsal side. The lobsters are located partly in the central portion/dorsal side, distal from the proposed inflow path, which makes transportation of the lobsters into the ammonoid shell unlikely. Additional evidence against transportation of the lobsters into the ammonoid shell comes from the fact that all the lobsters are visible in dorsal view, whereas more than one orientation would be expected in the case that the lobsters were washed into the shell. Moreover, transportation into the ammonoid shell would most likely result in breakage/disarticulation of the lobster specimens unlike the specimens presented here. In conclusion, we rule out transportation into the ammonoid.

### Molts or corpses?

Instead, the lobsters may have sought shelter to molt. Lobster molts may split along the median line and show misalignment of the abdomen and carapace with the carapace preserved on its lateral side [54] referred to as the ‘Lobster Open Molt Position’ [55]. A similar mode of molting for lobsters was mentioned previously [38], occurring as a result of lobsters molting on their side. Recently, it was shown that molted remains of erymid lobsters may also be preserved with the dorsal side up with or without a median split and with misalignment of the abdomen based on Middle Triassic lobsters from the Netherlands [56]. None of the abdomina and cephalothoraxes of the lobsters presented herein are misaligned and no median split can be observed, suggesting that the specimens are corpses rather than molts. However, it was suggested that some lobster molts can be preserved articulated, especially those preserved in quiet water conditions [38]. Additionally, it was stated that some lobsters molt in an upright position and may leave the carapace behind in its normal position, thus resembling a corpse [55]. Given that the sediments in the Posidonia Shale were deposited under relatively quiet water conditions and given the enclosed area of the lobsters within the ammonoid shell, these three specimens may either be interpreted as molts or corpses from this perspective.

Palinurids probably molted upright [55]. Although we favor another classification in which palinurids and eryonoids are classified within two different infraorders (Achelata and Polychelida, respectively) [57], eryonoids have been proposed to be related to palinurids as they both were listed as part of the infraorder Palinura [39]. If the latter is correct, eryonoids may have molted in the same fashion as palinurids. Additionally, Mertin [22] (p. 249) stated about molted palinurids that ‘both parts of palinurids [abdomen and carapace] have a skewed position relative to each other, both from the front and from the top [dorsal view]’. Thus, the specimens from this study might be interpreted as corpses because their abdomina and carapaces are not skewed. The assumption in both cases is that eryonoids and palinurids molted in the same manner. However, all eryonoids are extinct [57], so no direct proof exists as to their mode of molting. Moreover, as mentioned above, palinurids and eryonoids were also classified within two different infraorders, an interpretation which we favor. This implies that molting may have occurred sideways as well.

The lobster specimens are of similar length (see Table 1), especially the central and innermost lobsters. Lobster length is known to increase significantly per molt cycle in recent lobsters. The length of *Homarus americanus* was reported to increase by 11–12% in each molt cycle for a total length range of 140–223 mm [58]. Furthermore, a growth increment of 31.4% was noted for *H. americanus* with a cephalothorax length of 4.5 mm decreasing to 8.7% for a specimen with a carapace length of 128.5 mm [59]. Molt increments of 30–40 mm were noted for male specimens of *Palinurus mauritanicus* with a total length of 250–270 mm [60], which implies an increase of more than 10%. Increments per molt for *Nephrops* range from 3–12% although smaller and larger increments have been reported [61]. Thus, variation exists in the percentage of incremental increase among lobsters, but it is more than 10% in most cases. Hence, we infer that if the specimens represent three molts, then they must be from at least two individuals that molted in the same spot within the ammonoid, which we interpret to be very unlikely.

All three lobster specimens show a similar mode of preservation: their abdomina and cephalothoraxes are attached and the first pereiopods are nearly in the same place. The corpses of other decapods, notably shrimp and stomatopods decay fast [62], [63]. The cephalothorax in shrimp split from the abdomen after one to two weeks and disarticulation of the exoskeleton occurred after six weeks [62]. Even though the cuticle of the stomatopod *Neogonodactylus* was more robust than that of the shrimp [see 62], it still showed remarkably fast decay. Ruptures in the abdomen and/or in between the thorax and abdomen occurred after one week and disarticulation/fragmentation of the exoskeleton occurred after four weeks [63]. The same processes may be expected to occur on similar time scales for decapod molts. The results from these studies suggest that our lobster specimens may not be molts because that would imply that the animals molted at/around the same time in the same place, which is unlikely. The results of the studies on decay [62], [63] also suggest that the lobsters were most likely alive at the same time because of the very similar mode of preservation. If the lobsters were not alive at the same time then the modes of preservation are expected to differ, which we did not observe in the specimens.

Extant lobsters have been reported to eat their shedded exoskeleton to regain the lost calcium carbonate after molting when their mouth parts have hardened [64]. The female molt is reported to be mostly eaten by a male specimen of *Homarus americanus* after copulation during postmating cohabitation [65]. Assuming that specimens of the lobsters under study also ate the molt on a regular basis, the specimens cannot be molts.

In conclusion, we interpret the remains to be corpses rather than molts based on the completeness of the specimens in general, the preservation in dorsal position, the radial position of the lobsters within the body chamber with their tails close together, and a similar mode of preservation.

### Possible purposes of gregarious behavior

Because transportation of the lobsters inside the shell is unlikely (see above), the lobsters themselves must have entered the ammonoid shell while it was lying on the bottom of the ocean. This was possible because of the relatively small size of the lobsters in comparison to the aperture of the shell. Several scenarios might explain the presence of lobsters in the shell of *Harpoceras falciferum*: a) the ammonoid shell was an ideal spot to molt, b) the shell provided protection against predators, c) the decomposing soft body of the ammonoid provided a source of food, or d) the shell was used for long-term residency (inquilinism). In this case, these are examples of gregarious use of shelters, which have been reported for extant palinurids (e.g. [1], [5]). Interestingly, the formation of groups is enhanced by chemosensory cues [4].

Molting in a protected environment must have been beneficial to remaining protected for the time the new skeleton was not fully hardened. Molting of crustaceans in cephalopod shells has been suggested. Remains of a trilobite preserved in a Late Silurian nautiloid from the Czech Republic was interpreted to represent a molt [8]. Furthermore, the presence of a molted specimen of the lobster *Eryma dutertrei* was noted in a perisphinctid ammonoid shell from the Upper Jurassic (“Portlandian”) of the United Kingdom [36] and some molts of Triassic *Pseudopemphix* were found in internal molds of the body chamber of the nautiloid *Germanonautilus* [66]. More recently, crab molts were found in nautiloid shells from the upper Paleocene of Spain [46]. The specimens from this study may have sought shelter to molt, but had not yet molted (see above). As can be seen in Figure 3, the lobsters are located more than halfway within the body chamber toward the phragmocone and, thus, were out of direct sight of predators checking the aperture.

Protection unrelated to molting could be another reason to seek shelter in an ammonoid shell. The Posidonia Shale was not an environment in which many opportunities to shelter were available at the time of deposition. The most abundant shelters on the muddy ocean floor were ammonoid shells, especially specimens of the large *Harpoceras falciferum*. Other abundant ammonoids such as *Dactylioceras*, *Hildoceras*, and *Lytoceras* are smaller on average, and thus may have been impossible to access depending on the relative size of the decapod and ammonoid. Moreover, these shells were lighter and possibly more affected by bottom currents, and, thus, would represent an unstable shelter. To date, not a single decapod specimen has been found in shells of other ammonoid genera, whereas a few single decapod specimens have been found in *Harpoceras* shells from Dotternhausen (pers. observation). The decapods may have sought shelter to avoid predatory fish, as fish are known to be an important predator of extant lobsters (e.g. [67]), especially on small or juvenile lobsters [68]. Moreover, fishes may have influenced the evolution of crustaceans [69]. The presence of fish in mollusk and cephalopod shells is often proposed to be for protective reasons for the fish themselves (e.g. [11], [12], [70], [71], [72]), but fish also may have been actively hunting for prey hidden inside shells. There are numerous examples of predatory fish in ammonoid shells. For example, the presence of a macrosemiid fish, most likely a predatory fish, in a Late Jurassic (Kimmeridgian) ammonoid shell is known [72] and four Early Jurassic ammonoids from Germany and England contained one predatory fish (*Dapedium* sp. and *Pholidophorus* sp.) each with their head directed toward the phragmocone [37]. In one instance, the fish, *Dapedium* sp., apparently was stuck in a body chamber [37] (their Figs. 7–8). Three of the four specimens were also collected in the Posidonia Shale of Dotternhausen [37]. Predatory fish are known to prey on decapod crustaceans in the fossil record (e.g. [73]). Moreover, predatory fish were common in the waters of the Posidonia Shale [74], and, thus, were an immediate threat for decapods in open waters. We suggest that fish also may have been a threat for those decapods hiding in ammonoid shells. Given the small size of the lobsters presented in this study, they would be especially vulnerable to attacks by predatory fishes.

The lobsters may also have been searching for leftover tissue of the ammonoid inside the shell as the ammonoid specimen does not show any sign of the commonly found ‘ventral bite mark’ inflicted by a predator in the water column in this area [75], [76]. This could explain why there are as many as three individuals within this body chamber. The lobsters may have used chemoreceptive cues to discover the shell.

Decapods might also use the shell for storing food; the shell would, thus, have served for long-term residency [66]. Approximately 1% of the macroconchs of *Harpoceras falciferum* from Dotternhausen contains bivalve debris [35]. It was suggested that these bivalves were not the stomach remains from the ammonoid, but were probably ‘kitchen’ remains of an animal, probably a decapod, living in the shell [35]. No remains of possible leftover food from the lobsters were found in the body chamber of the ammonoid in our case, suggesting that storing food was probably not what happened here. On the other hand, since these particular lobsters have not been found outside ammonoid body chambers, they may have spent an important part of their time inside the ammonoid (see also below).

### Paleoecology and paleoenvironment

These particular, small lobsters have only been found in ammonoid body chambers so far. Not a single specimen is known that was not associated with an ammonoid shell after fifty years of collecting in Dotternhausen. The question then rises whether these decapods were preferentially preserved or whether the ammonoid shell was the place where they spent most of their time. A clue might come from other decapods from the Posidonia Shale. A specimen of *Palaeastacus*? sp. was found in a body chamber of *Harpoceras falciferum* from Dotternhausen [36], but isolated chelae of *Uncina posidoniae*, not associated with ammonoid body chambers, were found several meters stratigraphically below the other decapods [36]. Only one solitary large/adult *Eryma* sp. [74] has been found in the shales of Dotternhausen during all those years. Other localities in the Posidonia Shale in Germany also yielded decapods [77], [78], [79], [80], [81]: ?*Coleia theodorii*, *C. moorei*, *C. sinuata*, ?*Eryma* sp., *Glyphea grandichela*, *Proeryon giganteus*, *P. hartmanni* (* = P. banzensis*, *P. longiceps*, *P. macrophthalmus*), and *P. laticaudatus* (* = P. hauffi*), *Unica posidoniae*, and an undetermined specimen resembling an erymid [81]. None of these species has been reported from ammonoid body chambers, which suggests that the specimens from this study may have preferred the ammonoid shell as a shelter, but, moreover, may not have been preferentially preserved as numerous decapods have been found outside ammonoids.

The muddy bottom was not suitable for burrowing. Decapod burrows have not been found at the stratigraphic level of the studied specimens. The fact that the lobsters are present in the shell suggests that there was sufficient oxygen available above the sediment/water interface for at least some periods of time during deposition of the Posidonia Shale, despite the notion that these black shales would indicate oxygen depletion within this environment [31]. This is supported by the presence of other benthic organisms such as bivalves, brachiopods, and serpulids from the Posidonia Shale at Dotternhausen [33].

## Materials and Methods

The specimen containing the ammonoid and the three lobsters is stored in Oertijdmuseum De Groene Poort (Boxtel, The Netherlands) under museum number MAB k3166. The ammonoid and the lobsters were studied using standard microscopy and under low angle light. The latter appeared to be the best method to observe the details of the lobster specimens because the specimens were significantly flattened.
